# ECOMICS: A Web-Based Toolkit for Investigating the Biomolecular Web in Ecosystems Using a Trans-omics Approach

**DOI:** 10.1371/journal.pone.0030263

**Published:** 2012-02-01

**Authors:** Yoshiyuki Ogata, Eisuke Chikayama, Yusuke Morioka, R. Craig Everroad, Amiu Shino, Akihiro Matsushima, Hideaki Haruna, Shigeharu Moriya, Tetsuro Toyoda, Jun Kikuchi

**Affiliations:** 1 Plant Science Center, RIKEN, Yokohama, Kanagawa, Japan; 2 Graduate School of Nanobioscience, Yokohama City University, Yokohama, Kanagawa, Japan; 3 Advanced Science Institute, RIKEN, Wako, Saitama, Japan; 4 Bioinformatics and Systems Engineering Division, RIKEN, Yokohama, Kanagawa, Japan; 5 Biomass Engineering Program, RIKEN Cluster for Innovation, Wako, Saitama, Japan; 6 Graduate School of Bioagriculture Sciences, Nagoya University, Nagoya, Aichi, Japan; J. Craig Venter Institute, United States of America

## Abstract

Ecosystems can be conceptually thought of as interconnected environmental and metabolic systems, in which small molecules to macro-molecules interact through diverse networks. State-of-the-art technologies in post-genomic science offer ways to inspect and analyze this biomolecular web using omics-based approaches. Exploring useful genes and enzymes, as well as biomass resources responsible for anabolism and catabolism within ecosystems will contribute to a better understanding of environmental functions and their application to biotechnology. Here we present ECOMICS, a suite of web-based tools for ECosystem trans-OMICS investigation that target metagenomic, metatranscriptomic, and meta-metabolomic systems, including biomacromolecular mixtures derived from biomass. ECOMICS is made of four integrated webtools. E-class allows for the sequence-based taxonomic classification of eukaryotic and prokaryotic ribosomal data and the functional classification of selected enzymes. FT2B allows for the digital processing of NMR spectra for downstream metabolic or chemical phenotyping. Bm-Char allows for statistical assignment of specific compounds found in lignocellulose-based biomass, and HetMap is a data matrix generator and correlation calculator that can be applied to trans-omics datasets as analyzed by these and other web tools. This web suite is unique in that it allows for the monitoring of biomass metabolism in a particular environment, i.e., from macromolecular complexes (FT2DB and Bm-Char) to microbial composition and degradation (E-class), and makes possible the understanding of relationships between molecular and microbial elements (HetMap). This website is available to the public domain at: https://database.riken.jp/ecomics/.

## Introduction

Natural ecosystems can be conceptually thought of as interconnected environmental and metabolic systems. Humans and their activities affect and are a part of these ecosystems. For example, excessive nitrogen fertilizer may result in an alteration of soil, freshwater and marine ecosystems because of nitrate accumulation [1], [2]. In addition, other chemical changes due to anthropogenic activities like ocean acidification can alter microbial activity and composition [3]. Considering a more applied perspective of human activities within ecosystems, it is important to gain an understanding of natural ecology and its metabolic processes in various environments. From this perspective, biomass production is at the forefront of current research.

Biomass, which is produced by a diversity of living organisms and metabolic systems, has been harnessed by traditional human activities including agriculture, forestry, and fisheries. There currently however is considerable effort to transition from petrochemical-based raw materials, energy and manufacturing to a bio-based model; i.e. from oil-refineries to bio-refineries using newly applied biological methods [4], [5]. Similarly, identification of renewable enzymes to be used as reactive catalysts for chemical reactions leading to biomass production is a major focus [6], [7], [8], [9]. For example, it is important to monitor reactions and yields of intermediates as raw materials are converted to biomass products such as lignocelluloses in a quantitative manner in the chemical engineering field [10], [11], [12], [13], [14], [15], [16], [17].

Omics approaches have recently begun to be applied to investigations of ecosystem and biomass research. With this new field emerging, computer-aided technologies related to omics approaches are necessary for accumulating and processing experimental data. Further, handling tools are needed [18], [19], [20]. Based on the R platform, there are freely available tools to analyze omics datasets, such as the “ape” R package to visualize phylogenetic trees using genomic sequences. However, to our knowledge, there is no centralized group of freely available webtools that can accept and analyze heterogeneous omics datasets, including metagenomic and metabolomic data, and that quickly can produce output data both in numeric and visual format. We have reported on methodologies for analyzing metabolic dynamics in plant and bacterial systems [21], [22], [23], [24], [25], [26], [27], annotating metabolites [28], [29], [30], and revealing enzymatic networks [31], [32], [33]. Our results have shown how various combinations of genomic, proteomic, and metabolomic (including macromolecule for biomass) data can advance both ecosystem and applied research. Such a combination of multiple omics levels, here called “trans-omics”, can be applied to a wide range of biological systems from engineered to natural ecosystems.

**Figure 1:**
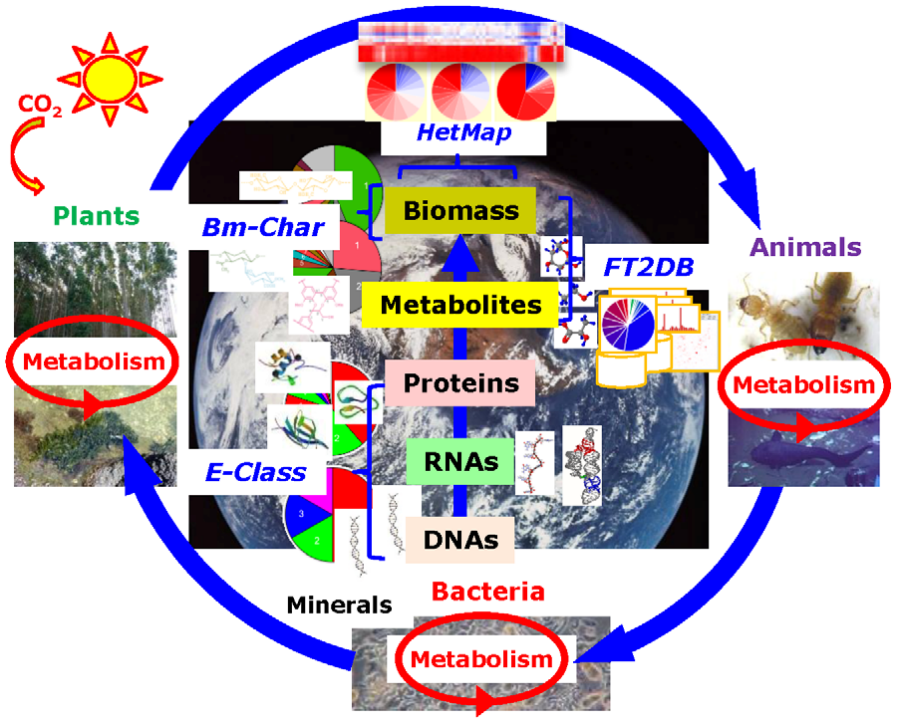
The ECOMICS schematic concept for analysis of relationships between the environment and omics datasets. Since the global ecosystem is composed of biodiversity in plant, animal and bacterial systems, our research target is not focused to single species, but accepts matrix datasets measured from complex systems. Experimental data should be comprised of a mixture of molecules from DNA to biomass. We developed four kinds of web tools and databases; the E-class web tool for taxonomic (metagenomic) classification based on prokaryotic and eukaryotic ribosomal sequences and for functional (enzymatic) classification based on sequential domains, FT2DB for the digitization of NMR spectra for downstream chemical (from metabolic to macromolecular) phenotyping, Bm-Char for the chemical (macromolecular biomass) assignment of lignocellulose components, and HetMap for identifying and viewing correlations between heterogeneous trans-omics data sets that are produced by such web tools.

In this paper, we introduce the ECOMICS web site as a source of information and tools useful for trans-omics approaches in ecosystem and biomass research (Figure 1). ECOMICS is made of the web tools including E-class for classification of ribosomal and enzyme sequence data, FT2B for the digital processing of NMR spectra for downstream analyses, Bm-Char for statistical assignment of specific compounds found in lignocellulose-based biomass, and HetMap for creating and visualizing data and correlation matrices derived from multi-omics datasets. These tools were designed as a unique web suite for analyzing elements included in environmental samples, e.g., sequential elements of metagenome and enzymes (E-class) and structural elements and compositions of metabolites and macromolecules (FT2DB and Bm-Char), and then associating these elements to reconstruct ecological relationships (HetMap). Namely, analysis of macromolecular complexity is a challenging field, but the ECOMICS web suite can uniquely calculate correlation coefficients (HetMap) not only within lignin-lignin or hemicellulose-acetyl signals, but also between lignocellulose components (Bm-Char) and the abundance and identity of degradation enzymes (E-class). The web site accepts heterogeneous omics datasets such as the combination of metagenome and metabolome data in common formats (FASTA format and NMR chemical shift data, respectively) and allows for the visualization of results through the internet. We believe that such simplicity leads to user-friendliness. This website is open to the public domain: https://database.riken.jp/ecomics/.

## Methods

### Web tools development

A common PC equipped with two CPUs (Quad Core Xeon 2.93 GHz), 24 GB memory and 2 HDs (146 GB) is used as a server machine for the ECOMICS web tools. Scripts and documents on the site were written using Java, Perl/CGI, JavaScript, and HTML. The operation of these tools was checked using Microsoft Windows (XP2, Vista, and 7), Macintosh (OS X), and Linux (Fedora 12) as operating systems and Microsoft Internet Explorer (version 8.0), FireFox (version 3.5.7), Google Chrome (version 8.0), Safari (version 5.0.3), and Opera (version 11.0) as Internet browsers.

### Example experimental data sets from an aquatic microcosm

To validate the utility of the E-class, FT2DB and HetMap tools, we prepared a small microcosm experiment to survey if any metabolite – community relationships could be revealed in an unbiased manner using the ECOMICS tools. Specifically, we expected community changes along the time course and were interested to see if we could also track concomitant changes in community metabolites. Several 1 L marine plankton microcosms were established using raw seawater from the mouth of the Nakarai River on Iriomote Island, Okinawa, Japan. At irregular intervals, 100 ml of microcosm samples were aseptically replaced with artificial seawater and nutrients (Daigo's SP and IMK, Nihon Seiyaku), and 25 ml of the removed water was filtered onto sterile 24 mm 0.22 µM Durapore filters (Millipore) in duplicate. Filters were vortexed in TE and nucleic acids were extracted from this solution as described in [21]. For PCR-DGGE the methods follow those of [34]. Methods for NMR spectroscopy [22], [26], [28], [35] have been previously described. Selected DGGE bands were excised from the gels, PCR reamplified using original primers without the GC-clamp, and purified PCR products were directly sequenced ABI 3130xl Genetic Analyzer with the BigDye Terminator v3.1 Cycle Sequencing Kit (Applied Biosystems, USA). The resulting edited sequences were submitted to “E-class”. For statistical analysis of DGGE - NMR data, a DGGE band-by-sample matrix was created for peak heights using Quantity One software (Bio-Rad laboratories Inc., Japan). NMR data was processed using “FT2DB”. A two-dimensional correlation map was calculated with “HetMap” as a symmetric matrix using Pearson's product-moment correlation coefficient in which an element at position (i, j) is defined as a correlation coefficient between the *i*th and *j*th positions in a set of 2D spectra of assigned metabolites and DGGE gel bands of identified bacteria.

### An example dataset obtained from a public database for detecting and classifying enzymes with the CBM domain

To check the performance of enzymatic function analysis for a large-scale query dataset using E-class, we obtained a dataset from the NCBI database (‘microbial46.protein.gpff’), composed of 84 402 peptide sequences.

### Example datasets for checking the performance of E-class

To compare the performance of E-class using different sequence databases, we set example datasets in which 100 sequences of 16S rRNA, 18S rRNA, and CBM were randomly selected from the complete database of 16S rRNA or the peptide sequences described above. These datasets were used for example queries against the different E-class databases to compare the speed of job completion for each reference database.

## Results

### E-class for taxonomic classification

**Figure 2:**
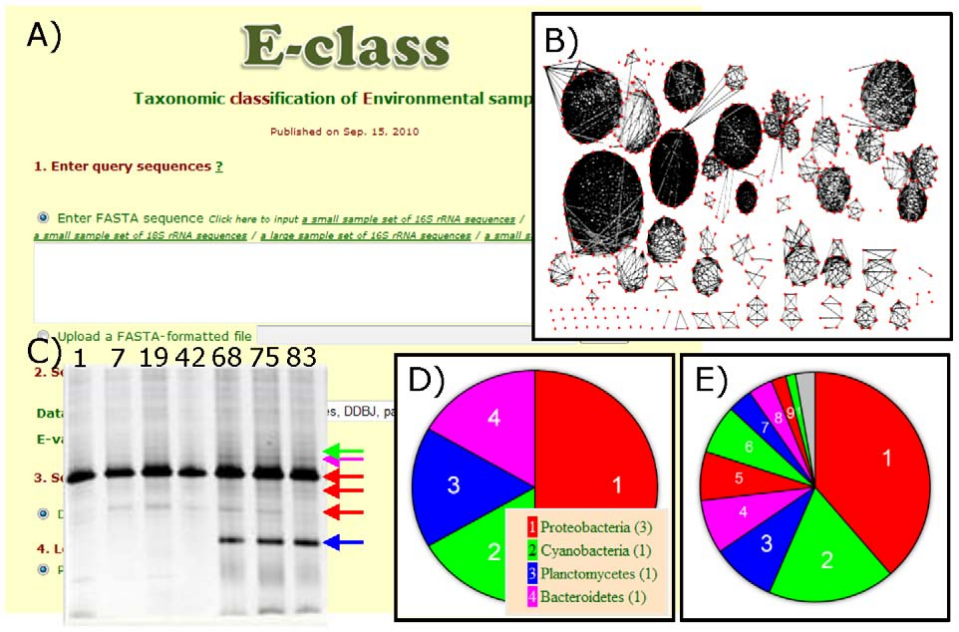
The E-class web tool to classify ribosomal and enzymatic sequences from environmental samples. (A) To query the E-class database, a several step process is used (see the Implementation section). (B) Network-based association between sequences of the E-class databases. A network module or group is composed of sequences and links between sequences representing sequence similarity. To reduce sizes of the ‘original’ databases, we selected one representative sequence from each identified module and set these as ‘modularized’ databases. (C) Our experimental example for a denaturing gradient gel electrophoreses (DGGE) analysis of a time series investigation of an Okinawa seawater microcosm experiment. Top numbers indicate sampling days for the microcosm experiments, and colored yellows are six DNA bands used for following E-class classification. (D) An example of this classification using our DGGE dataset as mentioned in Design and Implementation. (E) An example of a huge number of data, including 84 402 sequences, to the CBM sequence database, as mentioned in Design and Implementation.

E-class is a database and web tool for taxonomic classification of prokaryotic and eukaryotic DNA sequences and for the functional classification of enzymes using sequence domains found in environmental samples (Figure 2A; https://database.riken.jp/ecomics/eclass/). To classify query sequences, E-class utilizes a Basic Local Alignment Search Tool (BLAST) search of rRNA gene sequence databases obtained from public databases such as DDBJ (http://www.ddbj.nig.ac.jp/), NCBI (http://www.ncbi.nlm.nih.gov/), and Silva (http://www.arb-silva.de/) [36] and carbohydrate-binding module (CBM) sequences extracted from the RefSeq protein database (http://www.ncbi.nlm.nih.gov/RefSeq/).

This version of E-class provides a pie chart output of taxonomic and enzymatic classification. The freely available SVG viewer is required to depict a pie chart using Internet Explorer (http://www.adobe.com/svg/viewer/install/mainframed.html). Unlike other public databases which provide tools to search nucleotide or amino acid sequences, the BLAST searches implemented by E-class theoretically have no limitation in the number of input sequences. We show examples of taxonomic and enzymatic classification of query sequence datasets using E-class (Figure 2). Figure 2D represents a pie chart composed of taxonomic names at the phylum level that were assigned for six sequences obtained from a denaturing gradient gel electrophoreses [37] (DGGE) analysis of 16S rRNA gene fragments amplified from a time series investigation of an Okinawa seawater microcosm experiment (Figure 2C).

The steps for implementing a search are as follows (Figure 2A):

1. Enter query nucleotide sequences as FASTA-formatted text into the textbox of this step (to input sample data, click “Click here to input a sample sequence”) or select a file composed of such formatted text.
2. Select a database, i.e., 16S rRNA, 18S rRNA, and small subunit rRNA for prokaryotes, eukaryotes, and both taxonomical kingdoms, or CBM for enzymatic domains, respectively, (for the sample sequence, select 16S rRNA or small subunit rRNA) and enter the E-value (e.g., 1e-50) as a threshold for the BLAST search.
3. Select an output format (currently, only the pie chart function is available).
4. Select a level for taxonomic classification (phylum, class, order, or family; for the sample case, select “phylum”). If selecting a CBM database, this selection is ignored and query sequences are classified on the basis of the CBM category of Cazy (http://www.cazy.org/).
5. Click the “Submit” button to perform the analysis.

The BLAST search then starts. Once the search is completed, a pie chart of taxonomic or enzymatic classification is displayed along with a legend (Figure 2D and 2E). In Figure 2D, three of the six example sequences were assigned to the Proteobacteria, one was assigned to Cyanobacteria, one to Planctomycetes, and one to Bacteroidetes. Figure 2E shows the result of domain classification of CBM using an example dataset (‘microbial46.protein.gpff’), obtained from the NCBI FTP site. This query dataset is composed of 84 402 peptide sequences; 145 of which were detected as possessing a CBM domain: 56 sequences were classified as CBM2, 26 as CBM6, 13 as CBM51, 11 as CBM32, 10 as CBM16, 10 as CBM35, 5 as CBM20, 5 as CBM3, 3 as CBM47, 2 as CBM4, and single sequences as CBM10, CBM11, CBM23, and CBM25, respectively. The dataset has 143 sequences that include one or more CBM domains on the basis of their metadata, indicating that E-class detected two sequences with CBM domains but without the description of CBM in their metadata.

When a user queries thousands of sequences for classification, tens of hours may be required for the data to be processed using the common BLAST+ search. Thus we have added characteristics to the current version of E-class to improve the efficiency of the taxonomic and enzymatic classification. Users can select from the ‘original’ dataset and several subsets derived from the original dataset to query against. These subsets include one lacking partial sequences; these can be excluded from the BLAST search by selecting the ‘assignable’ or ‘curated’ database option. To reduce redundancy and execute a more rapid search, we also adopted a network module analysis [32], which assembles similar sequences into modules or groups (Figure 2B). We detected 5778, 4728, 23 998, and 134 local modules (including singletons), unconnected to other modules, for 16S rRNA, 18S rRNA, small subunit rRNA, and CBM, respectively. From each unique module, we selected the sequence that was connected to the most module members as the module representative in a ‘modularized’ database. Consequently, the size of each database was reduced to the above numbers from 222 054, 175 643, 262 092, and 4549 original sequences, respectively. These ‘modularized’ databases allow for much higher-throughput BLAST searches. These features are available for both 16S and 18S loci and CBM domains.

We executed several benchmarks to verify the performance of E-class. When an example dataset including 100 rRNA sequences was submitted, ‘assignable’, ‘curated’, and ‘modularized’ 16S rRNA databases required approximately 13, 10, and 1 minutes, respectively, indicating that the smaller-sized database enables more prompt retrieval. Second, the size of the query CBM dataset discussed above is comparable to that of a contig dataset obtained from a giga-sequencer and the output here shows the ability of E-class to handle such large datasets normally.

### FT2DB for chemical phenotyping

**Figure 3:**
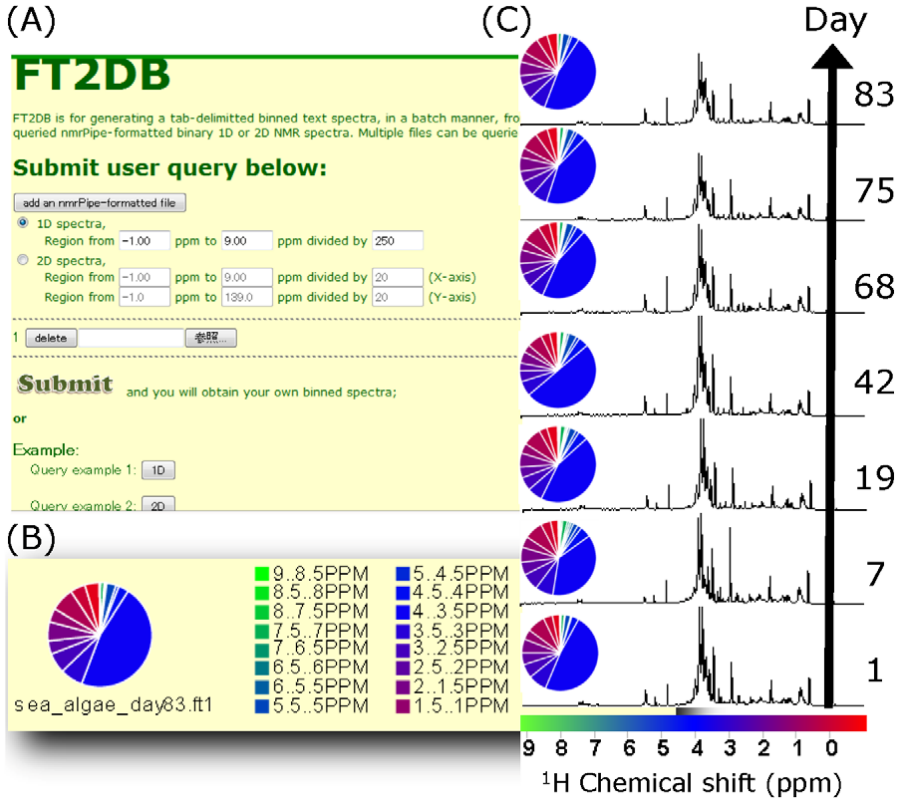
FT2DB for processing NMR spectra. (A) Screenshot of the FT2DB interface. This program allows for the simultaneous submission of several 1D or 2D NMR spectra and the user can specify the chemical shift range and the resolution of binning. (B) Representative output data from FT2DB. These data include pie charts for easy visualization and text data suitable for copying into a text editor for downstream analyses, such as with HetMap. (C) Resulting pie charts for the time series of 1D ^1^H NMR spectra of marine plankton. Chemical shifts are graded from red (−1 ppm) to green (9 ppm) and the fractions in spectrum intensity are shown in the pie charts. Spectra are labeled to the right with the sampling day. The raw NMR spectra are also shown. These examples are available at the website as are example 2D NMR spectra.

The package we offer is FT2DB (https://database.riken.jp/ecomics/chika/index2.html). This tool can digitize NMR spectra in a batch manner enabling users to easily edit spectra for construction of a bin database (Figure 3A). FT2DB is either a web-based service or a downloadable suite of programs that runs on MS Windows or Linux. FT2DB generates a tab-delimited text that contains all of the queried NMR spectra. The web-based service can handle both 1D and 2D queries. The standalone version of FT2DB contains the nmrbinDB1d program for 1D NMR spectra and the nmrbinDB2d program for 2D NMR spectra. These queries require “nmrPipe”-formatted NMR spectra as input files [38]. User can specify a region of interest and the number of bins for binning by each query. Pushing the “submit” button will generate binned spectra for the query. Additionally, for web-based queries, FT2DB will output a pie-chart representation of the overall distribution of user defined chemical shift regions. These regions are visualized by a red (upfield) to green (downfield) gradation for quick observation of differences between samples (Figure 3B). The standalone version of FT2DB package requires the Java Runtime Environment version 1.6 (Java 6) or later. The download file is uncompressed and stored locally to a user defined directory. The user should set a path to this directory, and the same path is set to the environmental variable FT2DB. For example, nmrbindb1d can convert ten nmrPipe-1D-formatted files to a text database file that contains one tab-delimited 1D NMR bin spectrum per line. In the nmrbinDB2d standalone package, the user will see a GUI window, which enables the use to view all converted bin spectra on 2D planes with positive bins as red and negative as blue. The data generated from a 2D query has a similar format as 1D results in both the web and standalone versions. To determine the position on the 2D plane, the header line should be consulted.

We have prepared a sample 1D ^1^H-NMR dataset (Figure 3C) from the same Okinawa microcosm time series used for the E-class example. Each pie-chart represents overall differences of distribution of 20 defined chemical shift regions described above. In this example, the day 42 sample exhibits a relatively large sugar signature (4–3.5 ppm) based on spectral intensities compared with the other samples. DGGE DNA bands (Figure 2C) revealed the simplest microbial community structure in this sample. This new method of pie-chart visualization quickly allows us to see overall metabolomic (NMR) changes concomitant with ecosystem (DGGE) changes. Such rapid observations based on the visualized output as presented here can then be used to inform more rigorous analytical approaches, in this case as detailed in section 4 (below).

A sample 2D ^1^H-^13^C dataset from two spectra of solubilized lignocelluloses from two grass species (family Poaceae) is also available at the FT2DB website.

### Bm-Char for biomass component assignment

**Figure 4:**
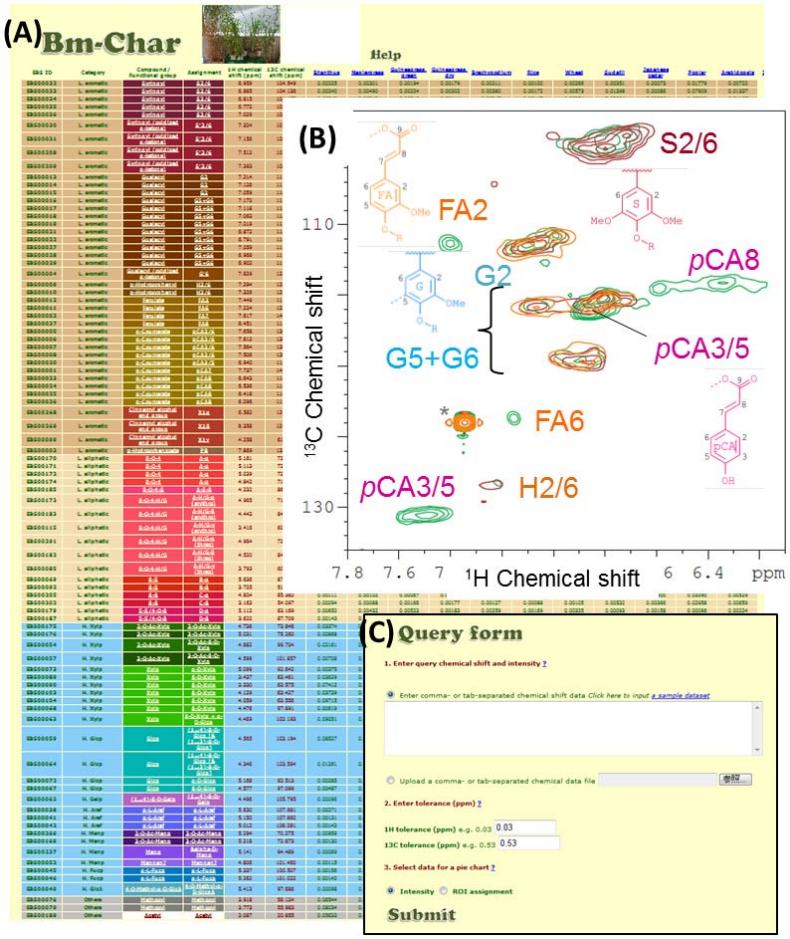
The Bm-Char diagrams. (A) A table of the Bm-Char main page, comprised of a data table displaying relationships between 88 chemical groups in 11 plant samples, 2D NMR signals for lignin and hemicellulose, and structures for components of lignocellulose. (B) Overlay of lignin aromatic region of 2D ^1^H-^13^C HSQC spectra of poplar (brown), Japanese cedar (light brown) and *Erianthus* sp. (green). Lignin signal assignments and their chemical structures are highlighted along with corresponding cross peaks. (C) The query form. See the main text for detail.

The Bm-Char webtool (Figure 4C; https://database.riken.jp/ecomics/biomass/) allows a user to retrieve biomass-related chemical components such as lignin and hemicelluloses from the chemical shift database we previously developed on the basis of 2D NMR spectral signals detected (Figure 4B) [39]. As of July 2011, the database is composed of 42 and 17 signals for aromatic and aliphatic sites of lignin, respectively, and, 26 signals for hemicellulosic-sites and three uncategorized sites. Figure 4A shows a table composed of these chemical signals (rows) of lignin and hemicellulose and 11 plant samples (columns); i.e., grasses: Erianthus, Napiergrass, Guineagrass (green and dry samples), Brachypodium, rice, and wheat, herbs: Arabidopsis, and trees: sudajii, Japanese cedar, and poplar. This table is available in the Bm-Char website. In the table, values represent intensities of individual signals in each plant sample. The rows are colorized according to the color chart to which we assigned chemical signals as described in the help page on the site; e.g., brown and red represent lignin signals and green, blue, and purple represent hemicellulose signals. These datasets can be used to retrieve query chemical shift data. Bm-Char accepts query datasets of ^1^H- and ^13^C-chemical shifts and if available, corresponding signal intensity values. These data are then output as a pie-chart showing matches to the database. Additionally output text files detailing the pie-chart composition and chemical shift assignments are available. In the example data, the pie-chart result is categorized according to items of ‘Detailed category’ described in the table on the Bm-Char website, including ‘Syringyl’, ‘Syringyl (oxidized alpha-ketone)’, ‘Guaiacyl’, ‘Guaiacyl (oxidized alpha-ketone), ‘p-Hydroxyphenyl’, ‘Ferulate’, ‘p-Coumarates’, ‘Cinnamyl alcohol end group’, and ‘p-Hydroxybenzoates’ for aromatic sites of lignin, ‘β-O-4’, ‘β-O-4-S’, ‘β-O-4-H/G’, ‘β-5’, ‘β-β’, and ‘5-5/4-O-β’ for aliphatic sites of lignin, ‘Acetylated xylopyranoside’, ‘Xylopyranoside’, ‘Xylopyranoside+glucopyranoside’, ‘Glucopyranoside’, ‘Galactopyranoside’, ‘Arabinofuranoside’, ‘Mannopyranoside’, ‘Fucopyranoside’, and ‘Methyl-glucuronic acid’ for hemicellulosic sites, and ‘Others’. The steps for making a query (Figure 4C) are as follows.

1. Input a query dataset formatted to include three successive columns without row or column labels; i.e., ^1^H chemical shift, ^13^C chemical shift, and signal intensity (if any). Bm-Char accepts a tab-, or comma-delimited text directly uploaded as input.
2. Input the tolerance of differences in ^1^H and ^13^C chemical shifts: the default values are 0.03 and 0.53, respectively.
3. Select the value type for the output pie chart. ‘Intensity’ or ‘Region of interest (ROI) assignment’ options are available. These represent the sum of ROI intensity values and the hit count of ROIs, respectively. If a query dataset contains only chemical shift data without intensity values, ‘ROI assignment’ is automatically selected.
4. Click the ‘Submit’ button.

A result page will load and display the output pie-chart, a chart legend, and a links to the downloadable text result files. In the near future, we will add information on chemical shifts of other biomass compounds that are or will become available from public databases or reports.

### HetMap correlation exploration for trans-omics data

**Figure 5:**
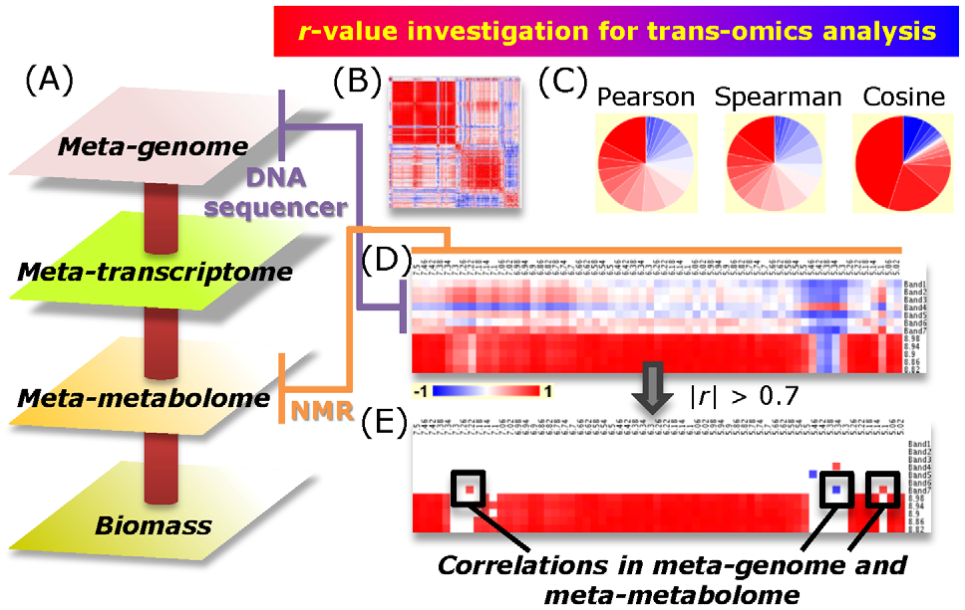
HetMap, a tool for performing a trans-omics analysis for multiple input data. Four input data files: meta-genomic, meta-transcriptomic, meta-metabolomic, and biomass data (A) from the same series of experiments for environmental samples are displayed in this figure. A user can obtain an image file that shows positive (red) and negative (blue) correlations among all the heterogeneous data that a user inputs (B). Example of *r*-value investigation using three kinds of correlation calculation (C, from left to right: Pearson, Spearman and cosine). Distribution of the correlation coefficient *r* is visualized in pie-chart with red (positive) to blue (negative) color gradation. Expanded region of 2D heat map image (|*r*|>0) derived from inputting DNA sequence (blue) and metabolites (green) data matrices of aquatic microcosm experiments (n = 7, Day 1, 7, 19, 42, 68, 75, and 83). From the number of samples included at a cutoff of *p*<0.05, we have chosen |*r*|>0.75 for visualizing significant correlation between DGGE and NMR data (E).

For revealing relationships between different omics levels (i.e genome, transcriptome, proteome, and metabolome data), many bioinformatics approaches have focused on the integration of multiple omics datasets [40], [41], [42]. HetMap (https://database.riken.jp/ecomics/chika/) is a convenient tool for easily generating a 2D heat map of correlations between heterogeneous types of data, such as metagenomic, metatranscriptomic, metabolomic, and biomass data. It simultaneously accepts up to four different types of omics or similar data. The principles of HetMap can be understood by first considering a standard correlation heat map. A correlation heat map is generated from a data matrix in which the correlation coefficient is calculated between two rows of data; then all pairwise comparisons between rows are calculated. A graphical representation of these coefficients is then produced showing either all, statistically significant or arbitrary cutoff values as different colors for positive and negative correlations. HetMap performs these functions, and allows users to query multiple data matrices simultaneously. Users can easily check combinations of their data samples without the need to build concatenated data files. To use HetMap, all input files should be tab-delimited text format. Data values are in rows, with the first column reserved as an ID column (e.g., for gene names) followed by the multiple data columns (e.g., for daily changes of the amount of transcripts). The number of columns must be the same for all of the input files. HetMap will output a correlation coefficient matrix that includes each pairwise combination for the input datasets, and generate a heatmap image for a quick visualization of the data. For example, when a metagenomic, a metatranscriptomic, a metabolomic and a biomass data file are input, HetMap generates a 2D heat map containing all the pairwise correlation coefficients between all sample IDs found in the four data files (Figure 5). The output image file uses red to indicate a positive correlation and blue to indicate a negative correlation. With HetMap it is possible to calculate correlation coefficients using Pearson, Spearman or cosine methods by either selecting the appropriate choice from the web-based drop-down menu or by specifying a string in a command line for the standalone software. In addition to the hetmap imager, a color key for correlation coefficients and a distribution of correlation coefficient *r*-values in pie-chart format are presented to help visualize the data. HetMap can be downloaded for Linux or MS Windows. The Java Runtime Environment version 1.6 (Java 6) or later is required.

In our example, the HetMap hetero-correlation output is shown (Figure 5B) correlating DGGE band intensity from Figure 2 with ^1^H-NMR spectra for each time point as processed by FT2DB. Based on the number of samples in our example, and selecting a cutoff of *p*<0.05 to mean significance, an |*r*|>0.75 equals to significant correlations among metabolites with DGGE bands (Figure 5E). Such correlations can then be investigated further as desired; in our case we identified the top 8 correlation pairs (including DGGE band – DGGE band and DGGE band – chemical shift correlations) and SpinAssign was utilized to putatively identify metabolites from NMR spectral bins of interest [28]. The top 8 correlation pairs that were 100% detected among 49 proton chemical shifts and that were assigned SpinAssign *p*-values>1.0e-10 (for correlations with metabolites) are provided in Table 1. A few observations are the clear positive relationship between a cyanobacteria (photoautotroph) and bacteroidetes (heterotroph), and the positive relationship between the cyanobacteria and a hydroxybutanoic acid. Relationships between Bacteroidetes and phytoplankton have been observed in natural systems [43], and this phylum is thought to play an important role in cycling organic carbon and other materials in aquatic ecosystems [44]. Cyanobacteria are also known to produce hydroxybutyrates; a group of compounds closely related to hydroxybutanoic acids and polyhydroxyalkanoates (PHAs) known for bioactive and biopolymer potential, respectively [45], [46]. Observations of patterns such as this using the tools provided here are a good validation of the potential for the ECOMICS web service, both in identifying relationships between taxa and in identifying compounds of interest.

**Table 1:** Top correlations (|*r*|>0.75) between DGGE bands and chemical shifts during time-course experiments (n = 7).

Band E-Class ID	Band or chemical shift (ppm)	Correlation *R* (n = 7)	Annotated metabolites (SpinAssin *p*-value)
*Cyanobacteria*	*Bacteroidetes*	1	-
*Proteobacteria*	3.22	0.893	Tyramine(0.73), β-Alanyl-N′-Histidine (0.057)
Unknown	*Cyanobacteria*	0.857	-
Unknown	*Bacteroidetes*	0.857	-
*Cyanobacteria*	1.02	0.857	Valine (2.1e-08)
*Bacteroidetes*	1.02	0.857	Valine (2.1e-08)
*Cyanobacteria*	1.34	0.821	2-Hydroxybutanoic acid (1.3e-09)
*Proteobacteria*	3.18	0.821	Tyrosine (0.96), β-Alanine (0.96), Choline (1.3e-09)

Although this HetMap tool is designed for analysis of trans-omics data, matrix correlations calculation can also be applied to self-correlation analysis. For example, a similar approach has been widely used in metabolite NMR data as Statistical Total Correlation SpectroscopY (STOCSY: [47]). Next we will demonstrate such application using our biomass NMR data sets.

### Application of HetMap tool to STOCSY-type analysis of biomass NMR data sets

**Figure 6:**
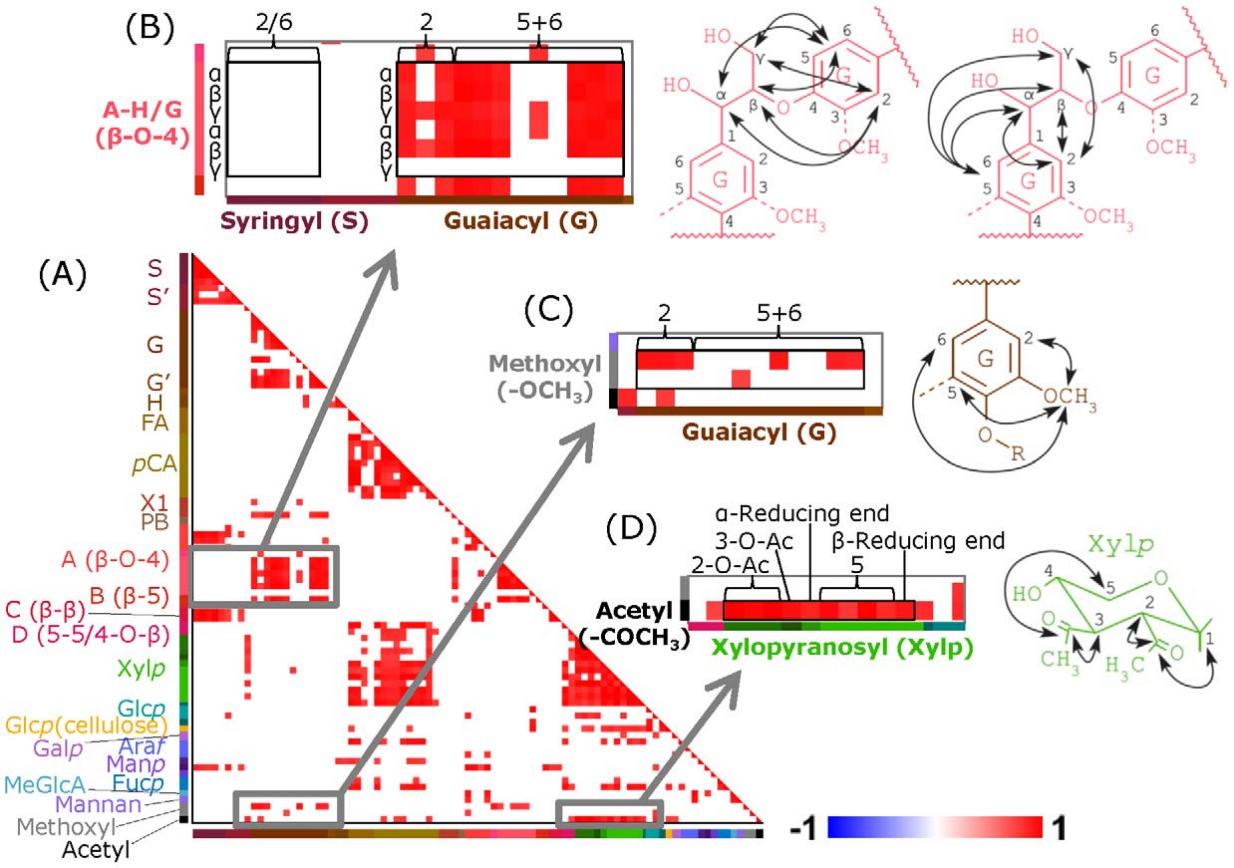
STOCSY-type analysis of biomass NMR spectra. (A) Self-correlation heatmap calculated by inputting 23 lignocellulose HSQC spectra into HetMap. A Pearson product-moment correlation coefficient with cut of |*r*|>0.75 was employed in this study. This heatmap shows ROI data matrices aligning from lignin aromatics, lignin, aliphatics to hemicelluloses sugars and methyl groups, and each molecules exhibited different colors along with vertical and horizontal bars. Expansion of lignin aromatics (guaiacyl, syringyl) versus aliphatics (β-O-4 hydroxyphenyl/guaiacyl) heatmap with showing calculated correlations in these chemical structures (B), as well as methoxy versus guaiacyl (C) and acetyl versus xylopyranose (D), respectively.

By adding twelve additional biomass NMR data sets (for a total of 23 HSQC spectra of lignocellulose components), we obtained a STOCSY-type self-correlation heatmap using HetMap (Figure 6). This heatmap shows showing ROI data matrices aligning lignin aromatics, lignin aliphatics, hemicellulose sugars and methyl groups (Figure 6A). Positive correlations indicate similar tendencies for functional groups to increase/decrease within the lignocellulose mixtures. Chemical groups within the same molecule or associated molecules can be expected to exhibit positive correlations. For example, the lignin aliphatics β-O-4-H/G exhibited high positive correlation with the guaiacyl region, whereas no correlation with the syringyl region was observed (Figure 6B). Methyl signals also showed reasonable correlations, such as methoxyl with guaiacyl (Figure 6C) and acetyl with xylopyranose. These “reasonable” correlations were calculated by HetMap tool using an arbitrary *r* cut off value = 0.75 with Pearson product-moment correlation coefficient.

## Discussion

ECOMICS has focused on the improvement of the techniques of omics analysis. First, to estimate the composition and dynamics of terrestrial and marine microorganisms, a metagenomic approach is useful. It is, however, difficult to grasp the extent of diversity of eukaryotes, including fungi and algae, which are abundant as biomass. Further, the extent of bacterial diversity is still largely uncharacterized and bacteria, even in low abundances, can drive essential biogeochemical cycles such as the global carbon cycle [48], [49]. Moreover, the composition and fluctuation of sequential domains in an environmental sample can show the functionality and its dynamics of enzymes in the sample. There are some stand-alone tools, e.g., the “ape” R package that performs taxonomic classification and depicts the results in the form of a phylogenic tree. However, there is, to our knowledge, no web-tool that can perform taxonomic and enzymatic classification of an environmental sample and quickly depict the result maps in forms of pie charts on a screen. Thus, we developed the E-class database for taxonomic classification of eukaryotes and bacteria using small subunits of ribosomal RNA (rRNA) gene fragments and for functional classification of enzymes using sequential domains in an environmental sample.

Next, to assess ecosystem processes on the basis of the chemical composition of organisms identified in the environment, we focused on the environmental metabolome using ^1^H-NMR. This has been the focus of an international consortium [50]. Physicochemical information obtained from NMR spectra shows high uniformity independent of device and compatibility of data through the standardization of conditions for individual organisms [51]. The NMR spectral approach is appropriate for chemical phenotyping, for quantitative analysis of phenotypes in a chemical composition, and for analysis of environmental fluctuations [52], [53], [54]. This compatibility originates from the COMET consortium of pharmaceutical companies, as well as the INTERMAP project [55], [56]. As an example of clear categorization of chemical phenotypes in habitats, NMR spectral data from fruit and vegetable juices were used to successfully identify their production locations [57]. As the switch to using biomass resources for bio-refinery applications occurs, an NMR spectrum of a cell wall provides a wealth of information on all wall components including high-resolution composition and structural “fingerprint” data [58]. Furthermore, NMR has remarkable potential for accurate quantification of individual chemical groups, even in complex metabolite mixtures [59], [60], [61]. Chemical phenotyping has been employed in various applications such as accurate quantitative determination of the intramolecular distribution of ^12^C and ^13^C in C_3_ and C_4_ plants [62]; quantification of ^1^H and ^2^H isotopomers of tree-ring cellulose [63]; characterization of dissolved organic nitrogen in the ocean, or water-soluble organic carbon in urban atmospheric aerosols [64], [65], [66]; and analysis of inorganic and organic complex molecular structures of plant biomass-derived black carbon in biomineralization [67]. FT2DB is a part of the ECOMICS system and thus designed to be in accordance with the other ECOMICS functions such as HetMap. FT2DB can generate a data matrix and this can immediately be copied and pasted to the input for HetMap. FT2DB generates pie charts for quick visualization. By digitizing the data using FT2DB and storing the data extracted from the environment, one can continuously collect and inspect environmental conditions.

Replacement of petrochemicals with bio-based compounds has made the focus of metabolite research in environmental samples [6], [17], [68], [69], [70]. The chemical structure of biomass products can affect differences in their degradability. However, information on the composition of biomass products such as lignocelluloses is limited in particular plant species and for particular chemical components, due to the difficulty of separation of lignin-carbohydrate complexes into each component (such as monosaccharides). Recently, advances in NMR spectral analysis revealed the composition of lignocellulosic products using a ball-milled sample without troublesome separation [39], [71], [72], [73], [74], [75]. Furthermore, NMR has the potential to monitor structural organization of supramolecular assembly of lignocellulose components by conventional 1D [76], [77], [78], as well as 2D and 3D magic angle spinning measurements [10]. Kim and Ralph [39] assigned chemical signals to lignocellulosic components including lignin aliphatic and aromatic sites and hemicellulosic sites. The BioMagResBank (BMRB) database [79] provides the important spectral and quantitative data derived from NMR spectroscopic investigations of biological macromolecules and metabolites, such as the lignocellulosic components mentioned above. It is useful for a lignocellulose researcher to retrieve query chemical signals obtained using NMR spectrometry and visualize the lignocellulosic composition of the signals. However, to our knowledge there is no web-tool allowing visualization of the output result. In order to provide a user-friendly approach for such visualization, we developed the Bm-Char web tool to characterize the composition of lignocellulosic components in an environmental sample on the basis of previous work [39].

HetMap is a simple correlation generator. It is very useful to quickly obtain an overview of the correlation as both text and image data, e.g., between enzymes and organisms associated with chemical reactions and products in a complex reaction field of environmental organisms. HetMap generates pie charts similarly to the other ECOMICS tools. General stand-alone tools such as MS-Excel or the R platform can also generate correlation matrices but typically require more time for data input, calculation, and generation of output visualizations. Thus HetMap is a convenient and rapid tool and is especially useful for depicting a heat map of correlations between or within omics datasets such as the transcriptome and the metabolome [80], [81].

Although many efforts have been made to develop omics approaches using various model organisms, recent advances in omics measurement methods and information technology allows for the development of more complex research approaches such as population omics [82]. This includes the systematic evaluation of biological interactions in natural environments. In particular, as research continues to advance the potential for bio-based manufacturing and energy over petro-based alternatives, we can expect a revolution in chemical engineering. The industrial revolution provided most human beings with access to a remarkable standard of living, yet this economic power has come at a cost to ecosystem function and viability. Conversely, pre-industrial economies did not allow for the general well-being of human populations, yet were more ecologically sustainable. By making tools available to the public domain that promote research on complicated biological information we hope to contribute to the next revolution in human economics; effective and sustainable human industry that draws upon the unused biomass, biodiversity and biochemistry found in natural ecosystems. We propose that omics research activity should be directed toward advancing a sustainable society that uses renewable bio-resources and promotes economic development but also maintains ecological health, hence the term ‘ECOMICS’; or ECOsystem OMICS.

The ECOMICS system is a useful web suite to reveal relationships between environmental samples across multiple omics levels. It is freely available and is open to the public domain: https://database.riken.jp/ecomics/. ECOMICS can accommodate trans-omics datasets such as biomolecular sequences (DNA, RNA, and amino acid) and metabolites (NMR chemical shift data). E-class can annotate extensive sequence datasets in a batch manner from several (e.g., DGGE bands) to more than 1 000 000 sequences (e.g., environmental metagenomic data). FT2DB digitizes NMR spectral data for correlation analysis between trans-omics datasets. Bm-Char identifies chemical signals of biomass-related compounds such as lignocellulose using a dataset derived from annotated NMR spectra. HetMap performs correlation analysis between datasets of sequences and metabolites annotated by E-class and Bm-Char and those obtained from FT2DB. All the ECOMICS tools quickly present easily visualized output information as pie charts.

Through the use of the web suite, a user can obtain information on the relationships between sequences (organisms and proteins) and chemical signals (metabolites) included in the user's environmental sample. For example, to evaluate the ability of an environment for degrading macromolecules, it is important to collect a various levels of omics data such as metagenome, metatranscriptome, and metabolome and to reconstruct their association network. Preliminarily, we pursued a process of cellulose degradation in a sludge environment in which cellulose was added. Selection of microorganisms related to the degradation and functional analyses of cellulose-degrading enzymes was performed using a next-generation sequencer and E-class. Metabolites derived from the cellulose were detected using solid-state NMR and FT2DB. We then attempted correlation analysis between these data using HetMap to reveal their direct associations. For systematic understanding of such complex environmental events, ECOMICS offers a single user-friendly platform that enables researchers to perform trans-omics approaches.
